# Preclinical evaluation of antimalarial activity of CPF-1 formulation as an alternative choice for the treatment of malaria

**DOI:** 10.1186/s12906-023-03973-2

**Published:** 2023-05-04

**Authors:** Prapaporn Chaniad, Tachpon Techarang, Arisara Phuwajaroanpong, Walaiporn Plirat, Prasit Na-Ek, Atthaphon Konyanee, Parnpen Viriyavejakul, Abdi Wira Septama, Chuchard Punsawad

**Affiliations:** 1 Department of Medical Sciences, School of Medicine, Walailak University, Nakhon Si Thammarat, 80160 Thailand; 2 Research Center in Tropical Pathobiology, Walailak University, Nakhon Si Thammarat, 80160 Thailand; 3 Department of Tropical Pathology, Faculty of Tropical Medicine, Mahidol University, Bangkok, 10400 Thailand; 4 Research Center for Pharmaceutical Ingredient and Traditional Medicine, Cibinong Science Center, National Research and Innovation Agency (BRIN), West Java, 16915 Indonesia

**Keywords:** New formulation, Antimalarial activity, Acute toxicity, *Plasmodium berghei*, *Plasmodium falciparum*, Kheaw Hom remedy

## Abstract

**Background:**

Kheaw Hom remedy is a traditional Thai medicine used to treat fever. Some plants used in the Kheaw Hom remedy show promising in vitro antimalarial activity. This study prepared novel formulations of plants from the Kheaw Hom remedy and evaluated their antimalarial and toxicological activities.

**Methods:**

Seven new formulations were prepared by combining at least three herbs of six selected plants from the Kheaw Hom remedy, namely *Mammea siamensis* Kosterm., *Mesua ferrea* L., *Dracaena loureiroi* Gagnep., *Pogostemon cablin* (Blanco) Benth., *Kaempferia galanga* L, and *Eupatorium stoechadosmum* Hance. In vitro antimalarial activities of each formulation’s aqueous and ethanolic extracts were evaluated using the parasite lactate dehydrogenase (pLDH) assay. Cytotoxicity in Vero and HepG2 cells was assessed using the MTT assay. An extract with good antimalarial potency and selectivity index (SI) was selected for in vivo antimalarial activity using Peter’s 4-day suppressive test and acute oral toxicity test in mice. In addition, bioactive compounds were identified using Gas chromatography-mass spectrometry (GC-MS) analysis.

**Results:**

Among the seven new formulations, ethanolic extracts of CPF-1 (Formulation 1) showed the highest activity with an IC_50_ value of 1.32 ± 0.66 µg/ml, followed by ethanolic extracts of Formulation 4 and Formulation 6 with an IC_50_ value of 1.52 ± 0.28 µg/ml and 2.48 ± 0.34 µg/ml, respectively. The highest SI values were obtained for the ethanolic extract of CPF-1 that was selected to confirm its in vivo antimalarial activity and toxicity. The results demonstrated a significant dose-dependent reduction in parasitemia. Maximum suppressive effect of the extract (72.01%) was observed at the highest dose administered (600 mg/kg). No significant toxicity was observed after the administration of 2000 mg/kg. Using GC-MS analysis, the most abundant compound in the ethanolic extract of CPF-1 was ethyl *p*-methoxycinnamate (14.32%), followed by 2-propenoic acid, 3-phenyl-, ethyl ester, (E)- (2.50%), and pentadecane (1.85%).

**Conclusion:**

The ethanolic extract of CPF-1 showed promising in vitro and in vivo antimalarial efficacy, with no toxic effects at a dose of 2000 mg/kg, suggesting that the ethanolic extract of CPF-1 may serves as a new herbal formulation for the treatment of malaria. Additional research is required for safety and clinical pharmacology studies.

## Background

Malaria continues to be one of the most serious and life-threatening infectious diseases caused by protozoan parasites of the genus *Plasmodium* [1]. According to a World Malaria Report in 2021, there were an estimated 241 million cases of malaria, with 627,000 malaria deaths in 2020 worldwide [2]. The majority of these deaths occur in high-risk populations, particularly in young children under the age of five in the sub-Saharan Africa region [2]. A considerable number of illnesses and fatalities have been reported in the World Health Organization (WHO) areas of Southeast Asia, the Eastern Mediterranean, the Western Pacific, and the Americas [2]. Currently, a major issue facing the world is the rise in drug resistance to numerous antimalarial medicine classes [3–5]. The WHO South-East Asia region reported that *P. falciparum* treatment failure rates for the first-line medication artemisinin-based combination therapy were 10% and as high as 93% in Thailand [2, 6]. This indicates the urgent need for new treatment options that are safer, more effective, and have a new mechanism of action. For centuries, natural products have been shown to be the primary source of medicine, used to develop effective drugs in the treatment of various diseases. Natural products such as plants and herbs contain plenty of bioactive components and are a precious source of pharmacological value [7]. In particular, quinine and artemisinin, the current standard drugs for the treatment of malaria, are derived from traditional medicines and plant extracts [8]. Therefore, plants and herbs offer an interesting choice for the research and development of antimalarial drugs.

Kheaw Hom remedy, a Thai traditional medicine, is used to treat people with high fever, especially those with chickenpox, measles, and herpes zoster [9]. This remedy consists of 18 Thai medicinal plants with cooling and bitter properties, which reduce blood toxin levels. Both oral consumption and topical use of Kheaw Hom are advised [9]. Previous studies have reported that this remedy has antibacterial, antiviral, anti-inflammatory, and antioxidant properties [10–14]. In addition, our previous study found that various medicinal plants from the Kheaw Hom Remedy showed promising antimalarial efficacy against the *Plasmodium falciparum* K1 strain with low toxicity to Vero cells, including *Mammea siamensis* Kosterm (*M. siamensis*), *Mesua ferrea* L. (*M. ferrea*), *Dracaena loureiroi* Gagnep. (*D. loureiroi*), *Pogostemon cablin* (Blanco) Benth. (*P. cablin*), *Kaempferia galanga* L. *(K. galanga)*, and *Eupatorium stoechadosmum* Hance. (*E. stoechadosmum*) (IC_50_ = 1.50 ± 0.03 µg/ml, 7.39 ± 0.89 µg/ml, 10.47 ± 0.01 µg/ml, 24.49 ± 0.01 µg/ml, 25.80 ± 0.63 µg/ml, and 28.15 ± 0.73 µg/ml, respectively) [15]. Moreover, the previous studies found that, the plants in this study possess several pharmacological properties as shown in Table 1. According to these several biological activities, the combination of *M. siamensis*, *M. ferrea*, *D. loureiroi*, *P. cablin*, *K. galangal*, and *E. stoechadosmum* may have therapeutic promise in malaria infection. Therefore, this has generated interest in developing new formulations of the Kheaw Hom Remedy, which can be used as an alternative medicine for the treatment of malaria.

**Table 1:** List of selected herbal plants, parts used and therapeutic properties

No	Plant species	Family	Plant part	Voucher number	Therapeutic Properties
1	*M. siamensis*	Guttiferae	Flowers	SMD 122006002	Treatment of heart disease, fever, antiproliferative, apoptotic, aromatase inhibitory, antimalarial activities and suppressive effects of NO production [16–20]
2	*M. ferrea*	Guttiferae	Flowers	SMD 122007001	Antibacterial, antiarthritic, antioxidant, immunomodulatory, antispasmodic, estrogenic, progestational, astyrosinase and elastase inhibitory activities, treatment of fever, astringent, antiinflammation, dysentery, and antityphoid disease [21, 22]
3	*D. loureiroi*	Dracaenaceae	Stem	SMD 096001007	Antinociceptive, antipyretic, antibacterial and antioxidant activities [23, 24]
4	*P. cablin*	Lamiaceae	Leaves	SMD 142031002	Treatment of colds, nausea, fever, headaches, and diarrhea [25]. Antipeptic ulcer, antimicrobial, antioxidative, anti-inflammatory, analgesic, antitumor, antidiabetic, immunoregulatory and antidiarrheal and antimalarial effects [25, 26]
5	*K. galanga*	Zingiberaceae	Rhizomes	SMD 288013007	Treatment of coughs and pectoral infections [27], anti-inflammatory, analgesic, antidiarrhoeal, vasorelaxant, sedative, antineoplastic, antimicrobial, antioxidant, antimalarial and cytotoxic activity [28]
6	*E. stoechadosmum*	Asteraceae	Leaves	SMD 072036002	Antibacterial activity [29]

Due to the rising antimalarial resistance, new formulations made from the selected medicinal plants would be a wise alternative to utilize. Therefore, this study aimed to prepare new formulations of the Kheaw Hom remedy from selected plants and evaluate their in vitro antimalarial and toxicological activities. Only one formulation with the highest SI value was selected to confirm the in vivo antimalarial activity and acute oral toxicity in a mouse model. Gas chromatography-mass spectrometry (GC-MS) was used to identify bioactive compounds in the selected formulations.

## Methods

### Plant material and management

Six selected plants with promising in vitro antimalarial activity from Kheaw Hom remedy, including *Mammea siamensis* Kosterm., *Mesua ferrea* L., *Dracaena loureiroi* Gagnep., *Pogostemon cablin* (Blanco) Benth., *Kaempferia galanga* L, and *Eupatorium stoechadosmum* Hance were selected and purchased at a Thai traditional drug store in Nakhon Si Thammarat Province, Thailand (Table 1). All plant materials were identified using botanical characteristic patterns by an expert botanist and specimens with voucher numbers of the plants were deposited at School of Medicine, Walailak University. After being cleaned of any dust or dead material, the plant material was dried in a hot air oven at 60 °C.

### Preparation of formulation and extraction

Seven new formulations were prepared by combining at least three herbs from six plants selected plants from the Kheaw Hom remedy (Table 2). Water extracts were prepared by reflux technique, whereas ethanol extracts were prepared by the maceration according to a protocol previously described [30]. The sixty grams of each formulation were soaked with 600 ml of 95% ethanol and macerated for 72 h at room temperature. In another set of experiments, powdered plants (60 g) of each formulation were extracted with 600 ml of distilled water under reflux for 2 h. Next, the solvent was filtered through gauze and Whatman No. 1 filter paper. The filtered extracts were dried using a rotary evaporator (Rotavapor®; Buchi, China). Finally, the dried plant extracts were then placed in screw-cap containers and stored at 4 °C until further use. The percentage yield of each extract was calculated using the following formula:

**Table 2:** Herbal compositions and percentage yield of ethanolic and aqueous extracts of seven new formulations

New formula	Plant composition	Mass of component	Yield (%)	
			Ethanolic solvent	Aqueous solvent
Formulation 1	*M. siamensis*	10 g	7.32	11.66
	*M. ferrea*	10 g		
	*D. loureiroi*	10 g		
	*P. cablin*	10 g		
	*K. galangal*	10 g		
	*E*. *stoechadosmum*	10 g		
Formulation 2	*M. siamensis*	12 g	8.35	11.41
	*M. ferrea*	12 g		
	*D. loureiroi*	12 g		
	*P. cablin* .	12 g		
	*K. galanga*	12 g		
Formulation 3	*M. siamensis*	15 g	10.21	11.72
	*M. ferrea*	15 g		
	*D. loureiroi*	15 g		
	*P. cablin*	15 g		
Formulation 4	*M. siamensis*	20 g	11.55	12.20
	*M. ferrea*	20 g		
	*D. loureiroi*	20 g		
Formulation 5	*M. ferrea*	12 g	8.38	12.47
	*D. loureiroi*	12 g		
	*P. cablin*	12 g		
	*K. galanga*	12 g		
	*E*. *stoechadosmum*	12 g		
Formulation 6	*D. loureiroi*	15 g	7.22	12.67
	*P. cablin*	15 g		
	*K. galanga*	15 g		
	*E*. *stoechadosmum*	15 g		
Formulation 7	*P. cablin*	20 g	2.92	14.60
	*K. galanga*	20 g		
	*E*. *stoechadosmum*	20 g		

$$\text{\% }\text{yield}\text{ =}\frac{\text{Weight of crude extract}\text{ }}{\text{Weight of initial plant powder}} \times 100$$

### In vitro cultivation and maintenance of *Plasmodium falciparum *K1 strain

*P. falciparum* K1 strain was cultured according to a previous study, with some modifications [31]. Venous blood (type O) was obtained from healthy adult volunteers who provided informed consent, and red blood cells were isolated for malaria culture. Briefly, the malarial parasite was grown in red blood cells and maintained in a complete medium (RPMI-1640) containing 2 mg/ml sodium bicarbonate, 10 µg/ml hypoxanthine (Sigma-Aldrich, New Delhi, India), 4.8 mg/ml HEPES (HiMedia, Mumbai, India), 0.5% Albumax II (Gibco, Waltham, MA USA), and 2.5 µg/ml gentamicin (Sigma-Aldrich, New Delhi, India). The culture was maintained in an incubator at 37 °C with 5% CO_2_. The percentage of parasitemia was monitored daily using Giemsa staining under a light microscope.

#### In vitro assessment of antimalarial activity

Both ethanolic and aqueous extracts of each formulation were tested for antimalarial properties using the parasite lactate dehydrogenase (pLDH) assay according to a previously described protocol [30, 32]. Briefly, the parasitized red blood cells at 2% hematocrit and 2% parasitemia were added to a 96-well cell culture plate. Then, 1 µL of novel formulation extracts was added to each well, with final concentrations ranging from 0.3 to 2,500 µg/ml. For further control, wells were filled with dimethyl sulfoxide (DMSO) (Merck, Darmstadt, Germany) and artesunate at concentrations ranging from 0.3 to 10.0 ng/ml (Sigma-Aldrich, New Delhi, India), as negative and positive controls, respectively. Non-infected red blood cells were used as blank controls. The plates were incubated in an incubator at 37 °C with 5% CO_2_ for 72 h, frozen three times at 20 °C, and thawed at 37 °C to obtain complete hemolysis. Next, 20 µL of the sample was transferred to a new 96-well cell culture plate, which contained 100 µL of Malstat reagent (Sigma-Aldrich, New Delhi, India), and 20 µL of nitroblue tetrazolium/phenazine ethosulfate solution (Sigma-Aldrich, New Delhi, India), and then stored in the dark for 60 min. Subsequently, 5% acetic acid (Merck, Darmstadt, Germany) was added to each well to stop the reaction. The absorbance of each well was measured at 650 nm wavelength using a microplate reader. Each assay was performed in triplicates. Finally, the percent inhibition and half maximal inhibitory concentration (IC_50_) were calculated using a nonlinear dose-response curve.

#### In vitro assessment of cytotoxicity

Both ethanolic and aqueous extracts of each formulation were tested for cytotoxic properties using a 3-(4,5-dimethylthiazol-2-yl)-2,5-diphenyltetrazolium bromide (MTT) assay according to a previously described protocol [15, 30]. Briefly, African green monkey kidney normal cells (Vero cells) and human hepatoma cell lines (HepG2 cells) were cultured in 96-well cell culture plates at a density of 1 × 10^5^ cells/ml and incubated in complete medium containing 10% fetal bovine serum at 37 °C with 5% CO_2_ for 24 h. After cell attachment, the novel formulation extracts at a concentration of 0–80 µg/ml were added to each well and then incubated at 37 °C for 48 h. DMSO and a two-fold dilution of doxorubicin were used as negative and positive controls, respectively. Untreated cells were used as blank controls. After incubation, the culture medium was discarded and replaced with 100 µL of fresh medium containing MTT reagent. The culture plates were then incubated at 37 °C in 5% CO_2_ for 3 h, followed by adding 50 µl of propanol, wrapping, and culturing for another 20 min at room temperature. Finally, the absorbance at 560 and 670 nm was measured using a microplate reader. All experiments were repeated thrice. The 50% cytotoxic concentration (CC_50_) was calculated from the dose-response curve.

#### Selectivity index (SI)

The SI, the value of the ratio between the toxic effect of the extract on cell lines and antimalarial activity on the parasite cells, is used to predict the sensitivity of the toxic effect and the efficacy of antimalarial activity in mouse models. A high SI value indicated that the extract exhibited potential with less toxicity and could be used for further study in mouse models. The high SI of the extracts toward Vero and HepG2 cells was used to determine which extract was a suitable candidate for in vivo evaluation, which was calculated using the following equation:

$$\mathrm{SI}\;=\;\frac{{\mathrm{CC}}_{50}}{{\mathrm{IC}}_{50}}$$

#### Gas chromatography-mass spectrometry analysis

The extract was analyzed on a Triple Quadrupole GC-MS/MS instrument (Agilent 7890 B GC system, Agilent Technologies, Santa Clara, CA, USA), equipped with an HP-5ms column (30 m × 0.25 mm, 0.25 μm) at a constant flow of 1.0 mL/min of helium. The detection used a mass-selective detector from Agilent 7000 C GC/MS Triple Quad (Agilent Technologies) with the setting of ionization energy at 70 eV and ion source temperature of 250 °C. The scan range was set to 33–600 amu. The column temperature was set at 60 °C for 2 min, and the temperature was increased to 150 °C at a rate of 10 °C/min, followed by 300 °C at 5 °C/min. The sample was injected at 1 µL with a split mode of split ratio 20:1. Finally, spectral data with ≥ 80% similarity, which compared to mass spectra of known compounds in the National Institute of Standards and Technology library, were selected to identify phytochemicals.

#### Animal experiments

Male ICR mice aged 6–8 weeks and weighing 25–30 g body weight were purchased from Nomura Siam International Co., Ltd., Pathumwan, Bangkok, Thailand. Mice were randomly grouped and acclimatized in cages for one week prior to the experiment. The experimental animal room was set to 22 °C, with a relative humidity of 50–60%. The lighting environment was set to 12 h of light and 12 h of darkness. The mice were allowed free access to pellets and clean water.

#### Four-day suppressive test (Peter’s test)

This test was used to measure the ability of the ethanolic extract of formulation 1 to inhibit the schizont stage of *P. berghei*-infected ICR mice. The 4-day suppressive test was performed according to previous studies with some modifications [33, 34]. The mice were divided into six groups of five mice each, as shown in Table 3. Briefly, mice were injected with 1 × 10^7^ red blood cells infected with *P. berghei* ANKA via the intraperitoneal route. After four hours post-infection, the negative control group received 200 µL of 7% Tween 80 solution, whereas the positive control group was given 6 mg/kg body weight artesunate or 25 mg/kg body weight chloroquine orally per day. In the experimental groups, the animals received daily oral doses of 200, 400, or 600 mg/kg body weight of the ethanolic extract of CPF-1 (Formulation 1). The dosage was used at low, moderate, and high doses of crude extract according to previous studies [34, 35]. The mice were administered each substance daily for 4 days (at 3, 24, 48, and 72 h post-infection). On the fourth day (96 h post-infection), thin blood films were obtained and stained with Giemsa solution and then observed under a light microscope at 100× magnification to determine the percentage of parasitemia. The percentage of parasitemia was calculated using the following formula:

**Table 3:** Group classifications and doses used in the 4-day suppressive test

Groups (*n* = 5/group)	Extract/drug	Dose (mg/kg)
Negative control	7% Tween 80	-
Positive control	Artesunate	6
Positive control	Chloroquine	25
Experimental group 1	Ethanolic extract of formulation 1	200
Experimental group 2	Ethanolic extract of formulation 1	400
Experimental group 3	Ethanolic extract of formulation 1	600

$${\% }\ \text{inhibition} = \frac{(\text{parasitemia of negative group}\text{ - }\text{parasitemia of treated group})}{(\text{parasitemia of negative group})} \times \text{100}$$

#### Acute toxicity test

Male ICR mice aged 6–8 weeks weighing 25–35 g were used according to the standard guidelines of the Organization for Economic Cooperation and Development (OECD) [36]. Mice were randomly divided into three groups: 2000 mg/kg body weight ethanolic CPF-1 extract-treated, 7% Tween 80-treated, and untreated groups. Prior to the trial, the mice were fasted for three hours and provided access to fresh water. To generate stock with a concentration of 2000 mg/kg, the ethanolic CPF-1 extract was dissolved in 7% Tween 80. The control mice received a single oral dose of 7% Tween 80 solution, whereas mice in the experimental group received a single oral dose of the ethanolic CPF-1 extract at 2000 mg/kg body weight. After the administration, the mice were observed for 30 min. Physical and behavioral changes, including rigidity, sleep, diarrhea, depression, abnormal secretion, and hair erection, were observed for 14 days. At the end of the experiment, the mice were administered a single dose of 60 mg/kg pentobarbital (Nembutal, Ceva, France). Blood was drawn for biochemical analysis using a cardiac puncture procedure. Furthermore, liver and kidney tissues were taken out and fixed with a 10% formalin solution for histological analysis.

#### Biochemical analysis

After plasma collection, biochemical measurements of liver and kidney function, including alanine aminotransferase (ALT), aspartate aminotransferase (AST), alkaline phosphatase (ALP), blood urea nitrogen (BUN), and creatinine (Cr), were performed using standard techniques (kinetic method for AST, ALT, ALP, and BUN, and fixed time method for Cr) in an AU480 chemistry analyzer (Beckman Coulter, USA).

#### Histopathological examination

Paraffin-embedded sections of liver and kidney tissues were subjected to standard histological processing according to previously described protocols [37, 38]. The sections were deparaffinized with xylene three times for 10 min each, rehydrated in a descending ethanol series for 5 min each, and stained with hematoxylin and eosin (H&E) solution. The tissue sections were further dehydrated by increasing the ethanol concentration, washed with xylene, and mounted on glass coverslips. Two impartial observers who were not informed of the experimental groups examined the stained slides under a light microscope to assess histopathological changes.

### Statistical analysis

The results are presented as mean ± SEM. IBM SPSS Statistics version 23.0 software (SPSS, IL, USA) was used for the statistical analysis. The *p*-value cutoff for the statistical difference was 0.05 (*p* < 0.05). Normal distribution was examined using the Kolmogorov-Smirnov goodness-of-fit test. One-way analysis of variance (ANOVA) was used to examine the statistical significance of percentage of parasitemia, percentage of suppression, food and water consumption, bodyweight, and liver and kidney biochemical parameters, and Tukey’s multiple comparison test was performed.

## Results

### Percent yield

Table 2 shows the percentage yields of the seven new formulations. The aqueous extracts produced higher yields than the ethanolic extracts. The aqueous extract of formulation 7 had the highest value of 14.60, whereas the ethanolic extract had the lowest value of 2.92.

### In vitro antimalarial activity and cytotoxicity

Of the seven formulations from both ethanolic and aqueous extracts, there were top three formulations that presented highly antimalarial activity, including the ethanolic extracts of CPF-1 (Formulation 1), Formulation 4, and Formulation 6 with an IC_50_ value of 1.32 ± 0.66 µg/ml, 1.52 ± 0.28 µg/ml, and 2.48 ± 0.34 µg/ml, respectively (Table 4). Notably, the ethanolic extracts of almost all formulations, except for the ethanolic extracts of Formulation 2, exhibited antimalarial activity greater than that of the aqueous extracts. For cytotoxic efficacy, most of the novel formulation extracts were nontoxic to Vero and HepG2 cells, except for the ethanolic extracts of Formulation 4 on Vero cells with a CC_50_ value of 37.01 ± 0.34 µg/ml (Table 4). According to the SI value, the ethanolic extract of CPF-1 showed an SI value of 60.10 for Vero cells and 51.84 for HepG2 cells, which was higher than those of the ethanolic extracts of Formulation 4 and Formulation 6. Based on these findings, the ethanolic extracts of CPF-1 demonstrated potential antimalarial efficacy against *P. falciparum* K1 strain and low toxicity to Vero and HepG2 cells. Therefore, the ethanolic extracts of CPF-1 were selected for further investigation in 4-day suppressive and acute toxicity tests in mice.

**Table 4:** In vitro antimalarial activity and cytotoxicity of seven new formulations

No	List		Ethanolic extract			Aqueous extract		
			**IC**_**50**_ (µg/ml)	**CC**_**50**_ (µg/ml)		**IC**_**50**_ (µg/ml)	**CC**_**50**_ (µg/ml)	
			*P. falciparum*	Vero cell	HepG2 cell	*P. falciparum*	Vero cell	HepG2 cell
1	Formulation 1		1.32 ± 0.66	79.34 ± 2.36 ^60.10^	68.43 ± 1.55 ^51.84^	35.72 ± 2.22	136.65 ± 13.15 ^3.83^	249.30 ± 34.30 ^6.98^
2	Formulation 2		15.69 ± 1.11	58.74 ± 1.04 ^3.74^	128.40 ± 0.40 ^8.18^	14.96 ± 1.11	192.10 ± 0.7 ^12.84^	176.15 ± 9.65 ^11.77^
3	Formulation 3		2.61 ± 0.25	50.02 ± 0.51 ^19.16^	51.38 ± 2.85 ^19.68^	25.41 ± 3.27	185.90 ± 8.8 ^7.32^	131.70 ± 4.50 ^5.18^
4	Formulation 4		1.52 ± 0.28	37.01 ± 0.34 ^24.35^	58.16 ± 7.76 ^38.26^	19.79 ± 2.23	268.2 ± 19.8 ^13.55^	114.80 ± 0.10 ^5.80^
5	Formulation 5		6.14 ± 1.09	83.28 ± 0.74 ^13.56^	70.56 ± 0.49 ^11.49^	22.92 ± 4.12	241.00 ± 19.3 ^10.51^	121.05 ± 9.05 ^5.28^
6	Formulation 6		2.48 ± 0.34	61.76 ± 1.18 ^24.90^	128.95 ± 8.05 ^52.00^	49.68 ± 0.45	130.65 ± 2.95 ^2.63^	224.65 ± 14.05 ^4.52^
7	Formulation 7		13.85 ± 1.83	88.22 ± 2.17 ^6.37^	95.956 ± 4.25 ^6.93^	55.75 ± 3.93	202.60 ± 17.6 ^3.63^	381.15 ± 17.85 ^6.84^
	Artesunate	IC_50_: 1.25 ± 0.52 ng/ml						
	Doxorubicin	CC_50_: 1.60 ± 0.23 µg/ml						

Data are presented as the mean ± SEM

IC_50_: 50% inhibition concentration, CC_50_: 50% cytotoxic concentration, Superscript values are selectivity index (SI)

### GC-MS analysis

**Fig. 1:**
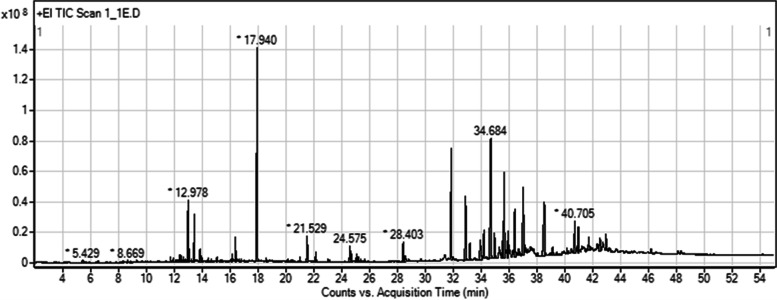
GC–MS chromatogram of ethanolic extract of CPF-1

A chromatogram of the ethanolic extract of CPF-1 is shown in Fig. 1. In total, 41 compounds were identified, as listed in Table 5. The most abundant compound was ethyl *p*-methoxycinnamate, with a retention time of 17.940, accounting for 14.32%, followed by 2-propenoic acid, 3-phenyl-, ethyl ester, (E)- (2.50%), pentadecane (1.85%), n-hexadecanoic acid (1.62%), patchouli alcohol (1.31%), and 9-octadecenamide, (Z)- (1.18%). In addition, the extract contained other compounds in a low quantity (less than 1%), such as oleic acid, β-patchoulene, cinnamic acid, and thymoquinone.

**Table 5:** Compounds identified in ethanolic extract of CPF-1 formulation

No	RT	Name of compound	Molecular formula	MW	Peak area (%)
1	4.311	Styrene	C_8_H_8_	104	0.06
2	6.532	Eucalyptol	C_10_H_18_O	154	0.06
3	8.669	endo-Borneol	C_10_H_18_O	154	0.08
5	9.324	2,4-Cycloheptadien-1-one, 2,6,6-trimethyl-	C_10_H_14_O	150	0.24
6	10.314	4-Hydroxy-3-methylacetophenone	C_9_H_10_O_2_	150	0.03
7	10.419	Carvacrol	C_10_H_14_O	150	0.02
8	10.814	3,5-Heptadienal, 2-ethylidene-6-methyl-	C_10_H_14_O	150	0.08
9	10.921	Thymoquinone	C_10_H_12_O_2_	164	0.04
10	11.718	Copaene	C_15_H_24_	204	0.17
11	11.844	β-patchoulene	C_15_H_24_	204	0.09
12	11.905	Tetradecane	C_14_H_30_	198	0.17
13	12.113	Cyperene	C_15_H_24_	204	0.13
14	12.264	Cinnamic acid	C_9_H_8_O_2_	148	0.15
15	12.395	Caryophyllene	C_15_H_24_	204	0.33
16	12.512	Ethanone, 1-(2-hydroxyphenyl)-	C_8_H_8_O_2_	136	0.32
17	12.686	Patchoulene	C_15_H_24_	204	0.06
18	12.788	Seychellene	C_15_H_24_	204	0.13
19	12.907	Humulene	C_15_H_24_	204	0.08
20	12.978	2-Propenoic acid, 3-phenyl-, ethyl ester, (E)-	C_11_H_12_O_2_	176	2.50
21	13.027	Alloaromadendrene	C_15_H_24_	204	1.01
22	13.220	Gamma.-Muurolene	C_15_H_24_	204	0.13
23	13.416	Pentadecane	C_15_H_32_	212	1.85
24	13.833	Flamenol	C_7_H_8_O_3_	140	0.91
25	13.949	Naphthalene, 1,2,3,5,6,8a-hexahydro-4,7-dimethyl-1-(1-methylethyl)-, (1 S-cis)-	C_15_H_24_	204	0.49
26	16.153	Ethyl *p*-methoxycinnamate	C_12_H_14_O_3_	206	0.35
27	16.248	Isoaromadendrene epoxide	C_15_H_24_O	220	0.10
28	16.376	Patchouli alcohol	C_15_H_26_O	222	1.31
29	16.640	Acetic acid, 3-hydroxy-6-isopropenyl-4,8a-dimethyl-1,2,3,5,6,7,8,8a-octahydronaphthalen-2-yl ester	C_17_H_26_O_3_	278	0.10
30	17.940	Ethyl *p*-methoxycinnamate	C_12_H_14_O_3_	206	14.32
31	20.131	4,4,8-Trimethyltricyclo[6.3.1.0(1,5)]dodecane-2,9-diol	C_15_H_26_O_2_	238	0.14
32	21.529	n-Hexadecanoic acid	C_16_H_32_O_2_	256	1.62
33	21.780	Naphthalene, decahydro-1,1,4a-trimethyl-6-methylene-5-(3-methyl-2,4-pentadienyl)-, [4aS-(4a.alpha.,5.alpha.,8a.beta.)]-	C_20_H_32_	272	0.07
34	22.129	Hexadecanoic acid, ethyl ester	C_18_H_36_O_2_	284	0.57
35	23.144	Kaur-16-ene	C_20_H_32_	272	0.08
36	24.575	9,12-Octadecadienoic acid (Z,Z)-	C_18_H_32_O_2_	280	0.90
37	24.667	Oleic acid	C_18_H_34_O_2_	282	0.83
38	25.053	Octadecanoic acid	C_18_H_36_O_2_	284	0.26
39	25.096	Linoleic acid ethyl ester	C_20_H_36_O_2_	308	0.37
40	25.200	Ethyl oleate	C_20_H_38_O_2_	310	0.25
41	28.403	Oleic acid amide	C_18_H_35_NO	281	1.18

*RT *Retention time, *MW *Molecular weight

#### Four-day suppressive test

The ethanolic extract of CPF-1 exhibited significant suppressive activity against *P. berghei* ANKA in a dose-dependent manner with 29.89%, 50.54%, and 72.01% suppression at doses of 200, 400, and 600 mg/kg doses, respectively, when compared to the negative control group (*p* < 0.001) (Table 6). The standard medication of artesunate at a dose of 6 mg/kg and chloroquine at a dose of 25 mg/kg exhibited 100% suppression of *P. berghei* parasitemia when compared to the negative control group, as well as significantly greater suppression than that of the other treated groups (*p* < 0.001) (Table 6).

**Table 6:** Percentage parasitemia and percentage suppression of *Plasmodium berghei* infected mice in 4-day suppressive test (Mean ± SEM)

Group	Dose (mg/ml)	% Parasitemia	% Suppression
Negative control	-	24.53 ± 1.94	-
Artesunate	6	00.00 ± 0.00	100.00 ^a, d, e, f^
Chloroquine	25	00.00 ± 0.00	100.00 ^a, d, e, f^
Ethanolic extract of CPF-1 (Treated groups)	200	17.20 ± 1.41	29.89 ± 5.76 ^a, b, c, e, f^
	400	12.13 ± 0.97	50.54 ± 3.95 ^a, b, c, d, f^
	600	6.87 ± 0.50	72.01 ± 2.04 ^a, b, c, d, e^

Data are presented as the mean ± SEM (*n* = 5 per group)

^a^ Compared with the negative control group receiving a mixture of 7% Tween 80 and 3% ethanol in distilled water, ^b^ compared with the positive control group receiving 6 mg/kg artesunate, ^c^ compared with the positive control group receiving 25 mg/kg chloroquine, ^d^ compared with 200 mg/kg extract, ^e^ compared with 400 mg/kg extract, ^f^ compared with 400 mg/kg extract

#### In vivo acute toxicity test

Daily behavioral and physical changes were observed throughout the 14-day follow-up period in mice administered a single dose of 2000 mg/kg CPF-1 ethanolic extract. Throughout the investigation, none of the mice showed overt signs of damage, such as hair erection, lacrimation, altered feeding activities, diarrhea, abnormal secretion, abnormal sleep, and excitement during the experiment. No mortality occurred during the first 24 h or over the next 14 days. These results demonstrated that the lethal dose of the extracts was greater than 2000 mg/kg body weight.

#### Effects of ethanolic extract of CPF-1 on liver and kidney functions

Biochemical parameters are summarized in Table 7. The levels of AST, ALT, and Cr in mice treated with 2000 mg/kg ethanolic extract of CPF-1 and 7% Tween 80 did not significantly differ from those of the untreated control group (*p* < 0.05). However, the levels of ALP in mice treated with 2000 mg/kg ethanolic extract of CPF-1 and untreated groups were significantly lower than the 7% Tween 80 group (*p* < 0.05). The levels of BUN in mice treated with ethanolic extract of CPF-1 were significantly increased when compared with those of the 7% Tween 80 group (*p* < 0.05).

**Table 7:** Effect of treatment on liver and kidney functions of mice in acute toxicity test

Group	Liver function test			
	AST (U/L)	ALT (U/L)		ALP (U/L)
Untreated control	25.40 ± 7.89	20.20 ± 1.93		93.20 ± 2.29 ^b^
7% Tween 80	32.80 ± 9.62	25.40 ± 1.94		112.00 ± 4.54 ^a, c^
Ethanolic extract of CPF-1	35.60 ± 9.09	24.00 ± 2.55		81.00 ± 6.27 ^b^
**Group**	**Kidney function test**			
	**BUN (mg/dL)**		**Cr (mg/dL)**	
Untreated control	19.60 ± 0.68		0.20 ± 0.00	
7% Tween 80	19.00 ± 0.00 ^c^		0.20 ± 0.01	
Ethanolic extract of CPF-1	21.00 ± 0.45 ^b^		0.22 ± 0.03	

Data are presented as the mean ± SEM (*n* = 5 per group)

^a^ Compared with the normal group, ^b^ Compared with the negative control group receiving a mixture of 7% Tween 80 and 3% ethanol in distilled water, ^c^ Compared with 2000 mg/kg extract

#### Histopathological analysis of the liver and kidney in the acute toxicity test

**Fig. 2:**
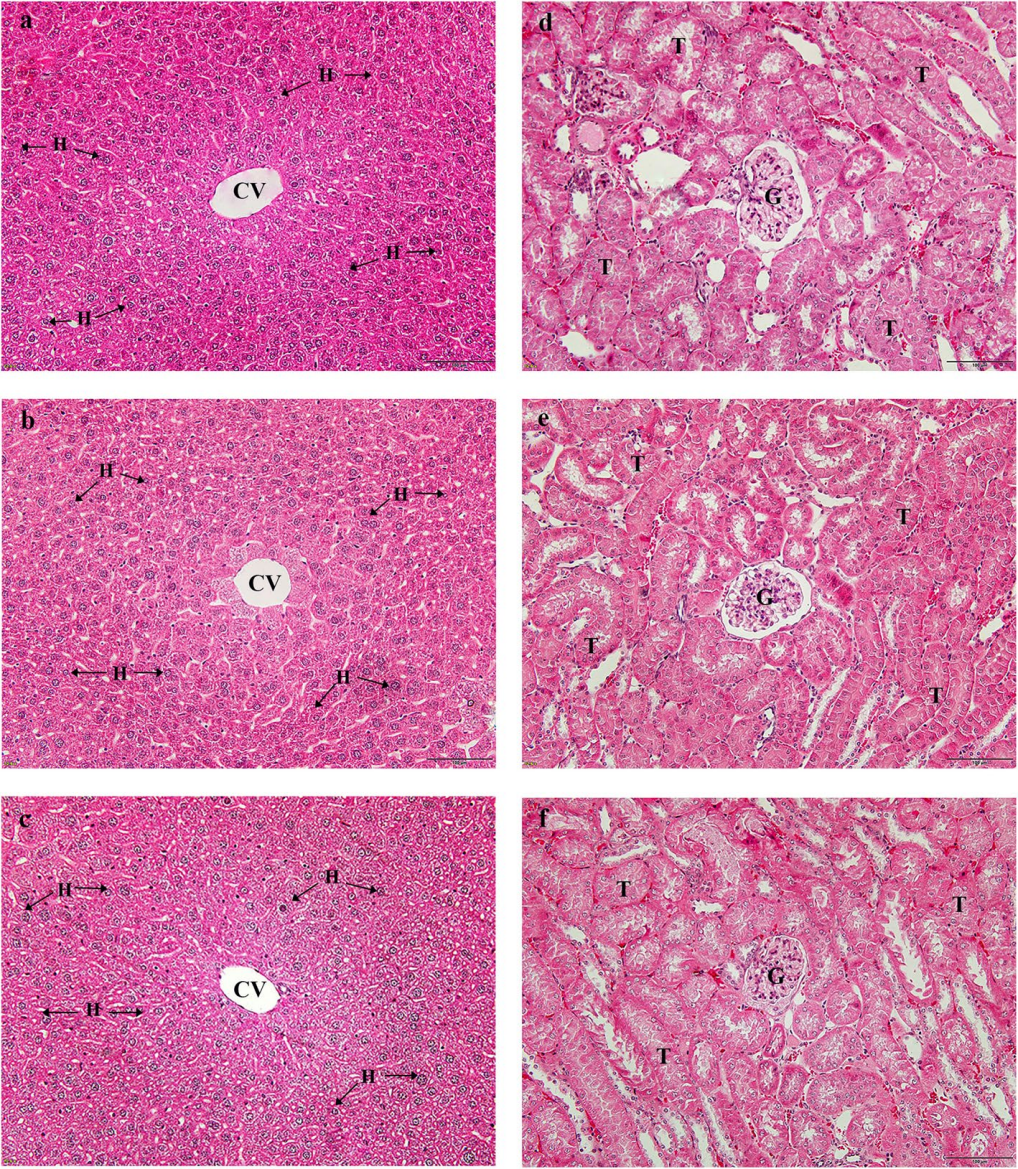
Histopathological examination in liver and kidney tissues from the control group (**a**) and (**d**), negative control group receiving a mixture of 7% Tween 80 and 3% ethanol in distilled water (**b**) and (**e**), and 2000 mg/kg extract (**c**) and (**f**). All images were photographed at 20X magnification. Scale bar = 20 μm. Central vein (CV), hepatocyte (H), tubules (T), and glomerulus (G)

Histopathological examination of the kidney and liver tissues is shown in Fig. 2. The liver tissues from the mice that received 2000 mg/kg of the extract showed normal liver structure with normal morphology of the central vein, hepatic sinusoid, and hepatocyte cells (Fig. 2c) when compared with the control group (Fig. 2a) and the negative control group (Fig. 2b). Examination of kidney tissue from mice that received 2000 mg/kg extract indicated normal histological structure of the glomerulus, Bowman’s capsule or glomerulus capsule, and renal tubule morphology. The kidney morphology of the mice that received the 2000 mg/kg extract (Fig. 2f) was not significantly different from that of the control group (Fig. 2d) and the negative control group (Fig. 2e).

## Discussion

Malaria elimination has become increasingly difficult since the emergence of antimalarial drug resistance. New tools such as vaccines or alternative treatments are required as soon as possible. The antimalarial properties of medicinal plants remain an available tool for drug research. Our previous study on the antimalarial activity of medicinal plants in the Kheaw Hom remedy found that six herbal ingredients possess antimalarial activity against *P. falciparum* [15]. As a result, new herbal formulations have been developed to improve the positive effects of the active ingredients. Seven formulae were investigated for in vitro antimalarial activity using the pLDH test. Enzymatic detection of pLDH was utilized as a marker of in vitro antimalarial activity [32]. According to previous research, the in vitro antimalarial activity of extracts can be categorized into four groups: highly active extracts with an IC_50_ of less than 5 µg/ml, promising activity at 5–15 µg/ml, moderate activity at 15–50 µg/ml, and inactivity at more than 50 µg/ml [39]. Our results showed that the top three new formulations, including the ethanolic extract of CPF-1, Formulation 4, and Formulation 6, revealed high antimalarial activity against the *P. falciparum* K1 strain with IC_50_ values of 1.32 ± 0.66 µg/ml, 1.52 ± 0.28 µg/ml, and 2.48 ± 0.34 µg/ml, respectively. The potential toxicity of the extracts was examined using Vero and HepG2 cells. The CC_50_ value was used to specify the potency of cytotoxicity in accordance with the categorization of cytotoxic cells. A nontoxic effect may be described as having a CC_50_ value greater than 50 µg/ml [40]. Both ethanolic extract of CPF-1 and Formulation 6 at a concentration of 80 µg/ml demonstrated low toxicity to both Vero and HepG2 cells with CC_50_ values of 79.34 ± 2.36 and 68.43 ± 1.55 µg/ml in ethanolic extract of CPF-1, and 61.76 ± 1.18 and 128.95 ± 8.05 µg/ml in ethanolic extract of Formulation 6, whereas ethanolic extract of Formulation 4 showed CC_50_ value of 37.01 ± 0.34 µg/ml on Vero cells. This indicates that the ethanolic extract of Formulation 4 is harmful to cells. The SI value is a highly important metric for utilizing extracts in subsequent studies [41]. A high SI suggests possible antimalarial activity and a safer course of treatment, whereas a low SI implies that it is probably toxic to the cells [42]. Based on the SI results, the ethanolic extracts of Formulation 4 and Formulation 6 showed SI values of 24.35 and 24.90, respectively, which were lower than that of the ethanolic extract of CPF-1, with an SI value of 60.10. Therefore, the ethanolic extract of CPF-1 was selected for further investigation in animal models because it exhibited the highest antimalarial properties and low toxicity.

In in vivo suppressive test, mice were used to examine the efficacy of the treatment against *Plasmodium* parasites [33]. Our results showed that animals treated with the ethanolic extract of CPF-1 at a dose of 600 mg/kg body weight had the highest suppression of *P. berghei* ANKA at 72.01%, followed by those with doses of 400 and 200 mg/kg body weight at 50.54 and 29.89%, respectively. This indicated that the ethanolic extract of CPF-1 at 600 mg/kg body weight has antimalarial properties. This may be caused by the presence of secondary metabolites in medicinal plants. A previous study on phytochemical screening in plant materials of Kheaw Hom remedy reported that the extracts of Kheaw Hom remedy strongly contained flavonoids, terpenoids, and alkaloids, followed by tannins, anthraquinones, saponins, and coumarins, respectively [15]. Therefore, the medicinal plants in the ethanolic extract of CPF-1, which was selected from the Kheaw Hom remedy, may also contain flavonoids, terpenoids, and alkaloids. These secondary metabolites with pharmacological activity may be responsible for the antimalarial activity [43, 44]. Flavonoids can prevent both chloroquine-sensitive and chloroquine-resistant strains of *P. falciparum* from growing intraerythrocytic phase by inhibiting fatty acid biosynthesis in the parasite [44, 45]. However, it may also function in the intraerythrocytic phase of the *Plasmodium* life cycle by preventing the influx of L-glutamine and myoinositol into infected erythrocytes [44]. Based on phytochemical profiling of the ethanolic extract of CPF-1 using GC-MS analysis, ethyl *p*-methoxycinnamate was found to be the major compound, which has been reported to have anti-inflammatory and anti-neoplastic effects [46, 47]. Among the various compounds, patchouli alcohol is one of the major components found in *P. cablin* Benth. and has been found to exert immunomodulatory effects by activating the mononuclear phagocytic system [48]. Our previous study reported that the ethanolic extract of *P. cablin* possesses in vitro and in vivo antimalarial properties without toxic effects [26]. Thymoquinone is a health-beneficial compound that is used against several illnesses, including infectious diseases, and has various therapeutic effects, including anti-inflammatory, antimicrobial, antioxidant, analgesic, and immunomodulatory effects [49]. For the mechanism of action of CPF-1, the compounds deposited in this formulation have been reported that, cinnamic acid hampered the production of ATP of parasite by inhibits transport of lactate, glucose, glycine, and sorbitol, which resulted in inhibit the growth of intraerythrocytic malaria parasites [50]. The antimalarial activity of the ethanolic extract of CPF-1 may have been produced from secondary metabolites deposited in the extract.

To assess the toxicity of the ethanolic extract of CPF-1, oral administration of the extracts at a single dose of 2000 mg/kg body weight was used to perform an acute toxicity test according to the OECD’s Globally Harmonized System of Classification [36]. No obvious symptoms, evidence of poisoning, or death were observed in the mice receiving 2000 mg/kg body weight of the ethanolic extract of CPF-1, which was identified as category 5 with a low acute toxicity hazard. Thus, the median lethal dose value or LD_50_ of the crude extracts was higher than 2000 mg/kg. Biochemical parameters and histopathological changes in the liver and kidneys were examined to confirm non-visible damage. Analysis of the biochemistry of liver and kidney function is crucial for determining the toxicological effects of xenobiotics [51, 52]. The enzyme markers, AST, ALT, and ALP, were determined to evaluate the abnormalities of the liver organ [53], and the levels of BUN and creatinine were investigated for kidney function. The change in BUN and creatinine levels in the blood circulation indicates the damage and aberration of kidney functions. The abnormality of BUN and creatinine levels might result from blood supply reduction to the kidney or urinary tract obstruction [54]. This study showed that the plasma levels of ALP in mice treated with the 7% Tween 80 solvent were higher than in the other groups, but plasma levels of AST, ALT, and Cr in both extract-treated and 7% Tween 80 groups were not different from the untreated control mice. This finding can imply that using 7% Tween 80 solvent to dissolve crude extract is something to be careful about. In the kidney function test, BUN levels in mice treated with the ethanolic extract of CPF-1 were significantly increased compared to the 7% Tween 80 group, but not significantly different when compared with the control group. This may be caused by a decrease in the renal processing of uric acid excretion, leading to an increase in uric acid content in extract-treated mice [55]. In addition, histopathological examination of the liver and kidney after treatment with the ethanolic extract CPF-1 did not show any abnormalities in these organs; therefore, this study indicated that oral administration of CPF-1 extract is not risky and is safe.

## Conclusions

This study reports that the ethanolic extract of CPF-1 demonstrated strong antimalarial efficacy against the *P. falciparum* K1 strain and *P. berghei* ANKA malaria parasites with no toxic effects both in vitro and in vivo assays, supporting the potential of this novel formulation as an alternative treatment for malaria. Clinical research is necessary to establish the safety and efficacy of novel formulations in humans.

## Data Availability

The data used to support the findings of this study have been included in this article. Additional files are available from the corresponding author upon request.

## Abbreviations

GC-MS: Gas chromatography-mass spectrometry
IC_50_: Half maximal inhibitory concentration
SI: Selectivity index
CC_50_: 50% cytotoxic concentration
